# Amphetamine-Like Analogues in Diabetes: Speeding towards Ketogenesis

**DOI:** 10.1155/2015/917869

**Published:** 2015-04-19

**Authors:** Natalia M. Branis, Steven D. Wittlin

**Affiliations:** Division of Endocrinology, Diabetes and Metabolism, University of Rochester School of Medicine and Dentistry, 601 Elmwood Avenue, P.O. Box 693, Rochester, NY 14642, USA

## Abstract

Obesity is common in patients with type 1 and type 2 diabetes. Amphetamine-like analogues comprise the most popular class of weight loss medications. We present a case of a 34-year-old African American female with a history of type 1 diabetes, dyslipidemia, and obesity who developed diabetic ketoacidosis (DKA) after starting Diethylpropion for the purpose of weight loss. Shortly after starting Diethylpropion, she developed nausea, vomiting, and periumbilical pain. Blood work revealed glucose of 718 mg/dL, pH 7.32 (7.35–7.45), bicarbonate 16 mmol/L (22–29 mmol/L), and anion gap 19 mmol/L (8–16 mmol/L). Urine analysis demonstrated large amount of ketones. She was hospitalized and successfully treated for DKA. Diethylpropion was discontinued. Amphetamine-like analogues administration leads to norepinephrine release from the lateral hypothalamus which results in the appetite suppression. Peripheral norepinephrine concentration rises as well. Norepinephrine stimulates adipocyte lipolysis and thereby increases nonesterified fatty acids (NEFA) availability. It promotes *β*-oxidation of NEFA to ketone bodies while decreasing metabolic clearance rate of ketones. In the setting of acute insulin deficiency these effects are augmented. Females are more sensitive to norepinephrine effects compared to males. In conclusion, amphetamine-like analogues lead to a release of norepinephrine which can result in a clinically significant ketosis, especially in the setting of insulin deficiency.

## 1. Introduction

As the obesity epidemic continues, the search for an effective weight loss solution is ongoing. Diet and exercise offer a variety of health benefits but they are effort-consuming and do not always guarantee success [1]. Among overweight and obese patients many suffer from type 1 diabetes and constitute a group in which weight loss can offer substantial health benefits [2]. Thus, many patients express interest in prescription weight loss medications.

Several classes of weight-reducing drugs are available in the United States, but the market is largely dominated by the amphetamine-like analogues [3]. Four preparations in this class include Phentermine, Phendimetrazine, Benzphetamine, and Diethylpropion. Out of the four, Phentermine is the most commonly prescribed. After the FDA issued a series of warnings about the competitors Sibutramine and Orlistat in 2010 the rate of Phentermine prescriptions rose from 82% to 98% of all weight loss medications [3]. Recently a combination of Phentermine/Topamax was approved by the FDA and became the first medication suitable for a long-term obesity management [4].

In randomized controlled trials, amphetamine-like analogues demonstrated significant efficacy with weight reduction of 10% or greater over a 12-week period in a majority of patients [5, 6]. Phentermine/Topamax combination achieved and sustained a 5–10% weight loss over a period of 28–56 weeks [7].

The absolute contraindications to the amphetamine-like analogues include a history of cardiovascular disease, uncontrolled hypertension, pulmonary hypertension, hyperthyroidism, glaucoma, use during or within 14 days following MAO inhibitor therapy, psychiatric illness with agitated state, pregnancy, and breast feeding [8]. No indication of its safety has been mentioned in regard to diabetes.

Our case report explores the validity of this recommendation. We describe a young woman, suffering from type 1 diabetes mellitus who developed diabetic ketoacidosis (DKA) shortly after the start of an amphetamine-like analogue.

## 2. Case Description

A 34-year-old African American female decided to seek medical attention with the goal of weight loss. Her past medical history included type 1 diabetes mellitus, dyslipidemia, and morbid obesity. She was diagnosed with type 1 diabetes at the age of 24 years, when she developed DKA. She had been using insulin pump for two years. She reported that she frequently skipped mealtime insulin boluses which resulted in poor diabetes control. Her last HbA1C was 9.2%. She denied a history of diabetic retinopathy, neuropathy, or nephropathy. She did not have episodes of DKA after the initial event that occurred at the time of diabetes diagnosis. Her medications included insulin Aspart and Rosuvastatin 5 mg daily.

She was prescribed Diethylpropion 75 mg daily by her primary medical doctor. In an attempt to accelerate weight loss she enrolled in Zumba classes. Ten days after starting Diethylpropion, the patient developed nausea, vomiting, and severe, cramping periumbilical abdominal pain. At that time, fingerstick blood glucose was ≈400 mg/dL (target 70–130 mg/dL). Patient changed the insulin pump site and administered several insulin boluses manually through the pump. These steps did not result in blood glucose improvement. She then administered an injection of a short-acting insulin. Nonetheless, significant hyperglycemia along with nausea, vomiting, and abdominal pain persisted.

On arrival to the hospital, patient's blood pressure was 127/72 mmHg, pulse 109 bpm, respiratory rate 16, O_2_ saturation 98% on room air, and BMI 38 kg/m^2^. Physical examination revealed an anxious young woman in moderate distress. Dry oral mucosa was noted. Tenderness to palpation in the periumbilical area was appreciated. There was no guarding or CVA tenderness. The rest of the physical examination was unremarkable. Blood work revealed plasma glucose of 718 mg/dL, pH 7.32 (7.35–7.45), bicarbonate 16 mmol/L (22–29 mmol/L), and anion gap 19 mmol/L (8–16 mmol/L). Urine analysis demonstrated large amount of ketones. Urine pregnancy test was negative. Chest X-ray and CT of abdomen and pelvis with contrast did not demonstrate any acute pathology.

The patient was admitted to the hospital and successfully treated for DKA. She was transitioned back to her insulin pump upon resolution of DKA. She continued to use it successfully. Diethylpropion was discontinued.

## 3. Discussion

Here we presented a case of a young woman with type 1 diabetes who developed DKA shortly after the initiation of amphetamine-like analogue for the purpose of weight loss.

Amphetamine-like analogues comprise the most popular class of weight loss medications due to their efficacy demonstrated in short- and long-term trials [3, 5–7]. The mechanism of their effect on weight is not entirely understood. It is thought that amphetamine-like analogues lead to release of norepinephrine and to a lesser degree serotonin and dopamine, in the hypothalamus. Norepinephrine stimulation of *β*_2_-adrenergic receptors within the hypothalamus results in the appetite suppression and subsequent weight loss [9].

Peripheral norepinephrine concentration rises as well. As demonstrated after Dextroamphetamine administration, plasma norepinephrine can rise up to 400 pg/mL, a level comparable to that achieved during mild physical activity [10, 11]. Cumulative effect on norepinephrine concentration is likely when amphetamine-type medications are given in the setting of acute illness or combined with activities leading to catecholamine release, such as exercise.

Norepinephrine exhibits a variety of effects in the peripheral tissues by affecting both *α*- and *β*-adrenergic receptors. In some patients stimulation of the *β*-receptors can result in offsetting the effect of *α*-stimulation, thus leading to tachycardia with normal blood pressure. In addition, norepinephrine promotes mobilization of lipids from white adipose tissue by stimulating lipolysis. Activation of *β*_3_-adrenergic receptors on adipocytes induces activation of adenylate cyclase and phosphorylation of hormone-sensitive lipase by a cAMP-dependent protein kinase. Hormone-sensitive lipase activation leads to a stepwise breakdown of stored triglycerides with formation of glycerol and nonesterified fatty acids (NEFA) [12]. In hepatocytes NEFA *β*-oxidation results in a formation of acetoacetate-CoA and acetyl-CoA, which are the primary substrates for ketogenesis [13].

The primary effect of norepinephrine on ketogenesis is mediated through increased substrate availability. As shown by Krentz et al., at high physiological concentrations, norepinephrine induces accelerated lipolysis and increases NEFA formation significantly [14]. Secondly, norepinephrine stimulates ketogenesis directly at the hepatocyte level. As reported by Keller et al. [15], norepinephrine infusion increased ketone bodies concentration to a greater degree when compared to NEFA concentration (155 ± 30 versus 57 ± 16%), suggesting direct hepatic ketogenic effect. The notion was further confirmed by Krentz et al., who observed that with equal NEFA availability increase in plasma norepinephrine level results in a greater ketone concentration than that induced by heparin infusion. The effect on hepatic ketogenesis can be even more pronounced when a decline in hepatic circulation, and therefore in substrate delivery, caused by norepinephrine is taken into consideration [14].

Among the ketone bodies, the most notable increase, in response to norepinephrine, is the *β*-hydroxybutyrate concentration [15]. Catecholamine-induced changes in ketone bodies' concentrations are caused not only by increased production but also by a decrease in their metabolic clearance rate. As reported by Keller et al., supraphysiological concentrations of norepinephrine resulted in a decline in ketone metabolic clearance rate by 35 ± 5%, *P* < 0.01 [15].

Gender differences in response to catecholamines were observed [16]. Matched elevation in norepinephrine level similar to that observed during moderate exercise resulted in higher glycerol release and NEFA concentration in women compared with men. This indicates a more robust lipolytic response to norepinephrine in females; also a higher rate of fat oxidation as demonstrated by lower nonprotein respiratory ratio was detected in women compared with men [16]. This can represent evolutionary adaptation in females to allow for a more efficient mobilization of adipose tissue energy stores in the setting of acute stress or illness.

Data on norepinephrine effects on plasma glucose and insulin concentration are controversial, with one study reporting no change [14] and another only modest increase [15]. Regardless, the achieved concentrations of insulin seem to be ineffective in preventing clinically significant ketosis [17, 18].

Insulinopenia augments ketogenic effects of norepinephrine as demonstrated by simultaneous infusion of norepinephrine and somatostatin [15]. In this setting ketone production increased dramatically and ketone clearance declined sharply. As a result, *β*-hydroxybutyrate concentration increased 12-fold, acetoacetate 4.3-fold, and acetone 4.1-fold during simultaneous norepinephrine and somatostatin infusion, degree of changes much more pronounced compared to norepinephrine infusion alone [15].

In summary, amphetamine-like analogues lead to an increase in norepinephrine concentration in the central nervous system as well as in the periphery. A cumulative effect on norepinephrine concentration is likely when amphetamine-type medications are administered during exercise or acute illness. Norepinephrine exhibits both direct and indirect ketogenic effects. It leads to augmented lipolysis with increased NEFA availability, stimulation of ketone bodies production, and decline in their clearance. Gender differences are observed with women being more sensitive to norepinephrine's lipolytic and ketogenic effects. Insulin deficiency augments the impact of norepinephrine even further.

According to current recommendations, no safety warning has been issued regarding the use of amphetamine-like analogues in patients with type 1 diabetes. Our review of available evidence on norepinephrine mechanism of action combined with our case report challenges this assumption. This class of medications may pose a risk of clinically significant ketosis in patients with type 1 diabetes and probably ketosis-prone type 2 diabetes.

For now, the safety of amphetamine-like analogues in patients with type 1 and ketosis-prone type 2 diabetes remains doubtful. They can result in clinically significant ketosis in these patients, especially in females, and should be used with caution. Patients and providers may consider the possibility of developing DKA when using amphetamine-like analogue.

## References

[1] Wing R. R., Phelan S. (2005). Long-term weight loss maintenance. *The American Journal of Clinical Nutrition*.

[2] Conway B., Miller R. G., Costacou T. (2009). Adiposity and mortality in type 1 diabetes. *International Journal of Obesity*.

[3] Block J. P., Choudhry N. K., Carpenter D. P. (2014). Time series analyses of the effect of FDA communications on use of prescription weight loss medications. *Obesity (Silver Spring)*.

[4] FDA (2012). *News Release*.

[5] Kang J. G., Park C.-Y., Kang J. H., Park Y.-W., Park S. W. (2010). Randomized controlled trial to investigate the effects of a newly developed formulation of phentermine diffuse-controlled release for obesity. *Diabetes, Obesity and Metabolism*.

[6] Kim K. K., Cho H.-J., Kang H.-C., Youn B.-B., Lee K.-R. (2006). Effects on weight reduction and safety of short-term phentermine administration in Korean obese people. *Yonsei Medical Journal*.

[7] Garvey W. T. (2013). Phentermine and topiramate extended-release: A new treatment for obesity and its role in a complications-centric approach to obesity medical management. *Expert Opinion on Drug Safety*.

[8] (1959). *Phentermine [Package Insert]*.

[9] Dietrich M. O., Horvath T. L. (2012). Limitations in anti-obesity drug development: the critical role of hunger-promoting neurons. *Nature Reviews Drug Discovery*.

[10] Nurnberger J. I., Simmons-Alling S., Kessler L. (1984). Separate mechanisms for behavioral, cardiovascular, and hormonal responses to dextroamphetamine in man. *Psychopharmacology*.

[11] Cryer P. E. (1980). Physiology and pathophysiology of the human sympathoadrenal neuroendocrine system. *The New England Journal of Medicine*.

[12] Nonogaki K. (2000). New insights into sympathetic regulation of glucose and fat metabolism. *Diabetologia*.

[13] Cotter D. G., Schugar R. C., Crawford P. A. (2013). Ketone body metabolism and cardiovascular disease. *American Journal of Physiology—Heart and Circulatory Physiology*.

[14] Krentz A. J., Freedman D., Greene R., McKinley M., Boyle P. J., Schade D. S. (1996). Differential effects of physiological versus pathophysiological plasma concentrations of epinephrine and norepinephrine on ketone body metabolism and hepatic portal blood flow in man. *Metabolism: Clinical and Experimental*.

[15] Keller U., Gerber P. P., Stauffacher W. (1984). Stimulatory effect of norepinephrine on ketogenesis in normal and insulin-deficient humans. *The American Journal of Physiology*.

[16] Horton T. J., Dow S., Armstrong M., Donahoo W. T. (2009). Greater systemic lipolysis in women compared with men during moderate-dose infusion of epinephrine and/or norepinephrine. *Journal of Applied Physiology*.

[17] Guy D. A., Sandoval D., Richardson M. A., Tate D., Flakoll P. J., Davis S. N. (2005). Differing physiological effects of epinephrine in type 1 diabetes and nondiabetic humans. *American Journal of Physiology—Endocrinology and Metabolism*.

[18] Avogaro A., Valerio A., Gnudi L. (1992). The effects of different plasma insulin concentrations on lipolytic and ketogenic responses to epinephrine in normal and Type 1 (insulin-dependent) diabetic humans. *Diabetologia*.

